# CircRNAs: a novel potential strategy to treat breast cancer

**DOI:** 10.3389/fimmu.2025.1563655

**Published:** 2025-03-19

**Authors:** Pangzhou Chen, Jinhui Zhang, Song Wu, Xiaoyu Zhang, Wen Zhou, Ziyun Guan, Hailin Tang

**Affiliations:** ^1^ The Sixth Affiliated Hospital, School of Medicine, South China University of Technology, Foshan, China; ^2^ State Key Laboratory of Oncology in South China, Guangdong Provincial Clinical Research Center for Cancer, Sun Yat-sen University Cancer Center, Guangzhou, China

**Keywords:** breast cancer, triple-negative breast cancer, CircRNAs, biomarkers, immune microenvironment

## Abstract

Breast cancer is among the most prevalent malignant tumors worldwide, with triple-negative breast cancer (TNBC) being the most aggressive subtype and lacking effective treatment options. Circular RNAs (circRNAs) are noncoding RNAs that play crucial roles in the development of tumors, including breast cancer. This article examines the progress of research on circRNAs in breast cancer, focusing on four main areas: 1) breast cancer epidemiology, classification, and treatment; 2) the structure, discovery process, characteristics, formation, and functions of circRNAs; 3) the expression, mechanisms, clinical relevance, and recent advances in the study of circRNAs in breast cancer cells and the immune microenvironment, particularly in TNBC; and 4) the challenges and future prospects of the use of circRNAs in BC research.

## 1 Introduction

Breast cancer remains a leading cause of cancer-related morbidity and mortality among women worldwide (1). According to the latest statistics, it accounts for a significant proportion of new cancer cases and deaths, underscoring the urgent need for effective prevention and treatment strategies (2). Among the various subtypes of breast cancer, triple-negative breast cancer (TNBC) is particularly notorious due to its aggressive nature and poor prognosis (3). TNBC is characterized by the absence of estrogen receptors, progesterone receptors, and human epidermal growth factor receptor 2 (HER2), which limits the options for targeted therapies that are effective in other breast cancer subtypes (4). Consequently, patients with TNBC often face a higher risk of recurrence and metastasis, making it imperative to explore novel therapeutic approaches and biomarkers (5).

Recent advances in molecular biology have shed light on the complex regulatory networks that govern cancer development and progression (6). One of the most intriguing discoveries in this context is the role of non-coding RNAs, particularly circular RNAs (circRNAs) (7). Unlike linear RNAs, circRNAs are characterized by their covalently closed circular structure, which confers them with increased stability and resistance to degradation (8). This unique feature enables circRNAs to function as molecular sponges for microRNAs (miRNAs), thereby modulating gene expression and influencing various cellular processes, including proliferation, apoptosis, and invasion (9).

The dysregulation of circRNAs has been implicated in numerous cancers, including breast cancer. Emerging evidence suggests that circRNAs can play multifaceted roles in tumor biology, acting as oncogenes or tumor suppressors depending on the context (10). For instance, certain circRNAs have been shown to promote tumor growth and metastasis by sequestering miRNAs that would otherwise inhibit oncogenic pathways. Conversely, other circRNAs may exert tumor-suppressive effects by promoting the expression of tumor suppressor genes or by interfering with pro-tumorigenic signaling pathways (11).

In the context of TNBC, circRNAs have garnered significant attention due to their potential to contribute to drug resistance, a major challenge in the treatment of this aggressive subtype (6). Studies have demonstrated that circRNAs can modulate the response of TNBC cells to chemotherapy and targeted therapies by regulating key signaling pathways, such as the PI3K/AKT/mTOR pathway (12). Furthermore, circRNAs may influence the tumor microenvironment (TME) by interacting with various immune cells, thereby shaping the immune landscape of the tumor and affecting its response to immunotherapy (13).

Despite the promising insights into the role of circRNAs in breast cancer, several challenges remain. The mechanisms by which circRNAs exert their effects on tumor biology are still not fully understood, and the functional diversity of circRNAs necessitates a comprehensive investigation into their specific roles in different breast cancer subtypes. Additionally, the potential of circRNAs as biomarkers for diagnosis, prognosis, and therapeutic response remains to be explored in clinical settings.

This review aims to provide a comprehensive overview of the current understanding of circRNAs in breast cancer, with a particular focus on TNBC. We will discuss the biogenesis, cellular localization, and degradation mechanisms of circRNAs, as well as their interactions with miRNAs and other molecular players in the tumor microenvironment. Furthermore, we will highlight the emerging evidence linking circRNAs to drug resistance and their potential as therapeutic targets. By synthesizing the existing literature, we hope to elucidate the multifaceted roles of circRNAs in breast cancer and pave the way for future research aimed at harnessing their potential for clinical application. Ultimately, understanding the intricate interplay between circRNAs and breast cancer biology could lead to innovative strategies for improving patient outcomes and advancing the field of cancer therapeutics.

## 2 Breast cancer

### 2.1 Epidemiology of breast cancer

Breast cancer arises from the carcinogenesis of breast epithelial tissue and has become one of the most prevalent malignant tumors worldwide, with an incidence rate second only to that of lung cancer (1) (**Table 1**). In 2022, breast cancer became the fifth leading cause of cancer-related deaths globally, accounting for 665,684 fatalities—a significantly greater number of deaths than other gynecological malignancies, such as cervical and ovarian cancers. Additionally, approximately 2.31 million women worldwide were newly diagnosed with breast cancer. The global incidence of breast cancer continues to increase at an annual rate of approximately 3.1% (2) (**Table 2**). In addition, the growth rate of breast cancer in Chinese women is much greater than that in the rest of the world (14). Furthermore, the age of breast cancer onset is decreasing. Combined with factors such as societal development, population growth, and an aging population, the economic burden of cancer on women worldwide has been steadily increasing (15).

**Table 1:** The top ten cancers with estimated incidence rates and mortality rates in the female population, United States, 2024.

	Estimated new cases		Estimated deaths
Breast cancer	310,720	Lung cancer/Bronchial carcinoma	59,280
Lung cancer/Bronchial carcinoma	118,270	Breast cancer	42,250
Endometrial carcinoma	67,880	Pancreatic cancer	24,480
Colon cancer	52,380	Colon cancer/rectum cancer	24,310
Melanoma of the skin	41,470	Endometrial carcinoma	13,250
Non-Hodgkin lymphoma	36,030	Ovarian cancer	12,740
Pancreatic cancer	31,910	Liver cancer/Intrahepatic cholangiocarcinoma	10,720
Thyroid carcinoma	31,520	Non-Hodgkin lymphoma	8,360
Renal carcinoma/Carcinoma of renal pelvis	29,230	Brain and other nervous system tumors	8,070
Bladder cancer	20,120	Myeloma	5,520

**Table 2:** New cases of the top five cancers with the highest incidence in 2022.

cancer	cases	rate
Lung cancer	2,480,301	12.40%
Female breast cancer	2,308,897	11.60%
Colorectum cancer	1,926,118	9.60%
Prostate cancer	1,466,680	7.30%
Stomach cancer	968,350	4.90%
others	10,814,465	54.20%

### 2.2 Classification, immune microenvironment and treatment of breast cancer

There are three main subtypes of breast cancer: ER/PR-overexpressing breast cancer (accounting for approximately 60% of cases), HER2-overexpressing breast cancer (approximately 20%), and triple-negative breast cancer (TNBC) (approximately 20%) (3). These subtypes differ in terms of gene expression, progression rates, metastasis potential, treatment approaches, and prognoses (4). The immune microenvironment of breast cancer is a complex network of immune cells, signaling molecules, and tumor components that play critical roles in tumor progression and immune evasion (16). The microenvironment includes various immune cells, such as T cells, macrophages, dendritic cells, and regulatory T cells, each of which contributes to either promoting or inhibiting the immune response. Tumor cells often employ immune checkpoint pathways, such as PD-L1, and secrete immunosuppressive factors such as TGF-β to evade immune detection (17). The characteristics of the immune microenvironment are closely linked to tumor aggressiveness, prognosis, and response to treatment, making the microenvironment a crucial focus for developing targeted immunotherapies (18). Breast cancer mortality rates have decreased due to early screening, detection, diagnosis, and improved treatment methods. However, TNBC is still associated with a poor prognosis and limited treatment options (19). Drug resistance, along with the potential for recurrence and metastasis, further complicates treatment (5). Recent evidence suggests that circRNAs may indirectly influence cancer progression through regulating gene expression (7). In particular, multiple circRNAs linked to breast cancer progression have been identified, which may serve as biomarkers and therapeutic targets for triple-negative breast cancer (6).

## 3 CircRNAs

### 3.1 Structure and classification of CircRNAs

CircRNAs are noncoding, closed loop RNA molecules without 3’ and 5’ end caps that connect different splicing domains through covalent bonds. They have a highly stable structure and are resistant to degradation by nucleases (8). CircRNAs can be categorized into three types: exonic circRNAs (ecircRNAs), which contain only exon sequences; circular intronic RNAs (ciRNAs), which contain only intron sequences; and exon−intron circRNAs (EIciRNAs), which contain both exon and intron sequences (9). While most circRNAs are located in the cytoplasm, a few consisting solely of intron sequences are found in the nucleus (10).

### 3.2 Discovery process of CircRNAs

**Figure 1:**
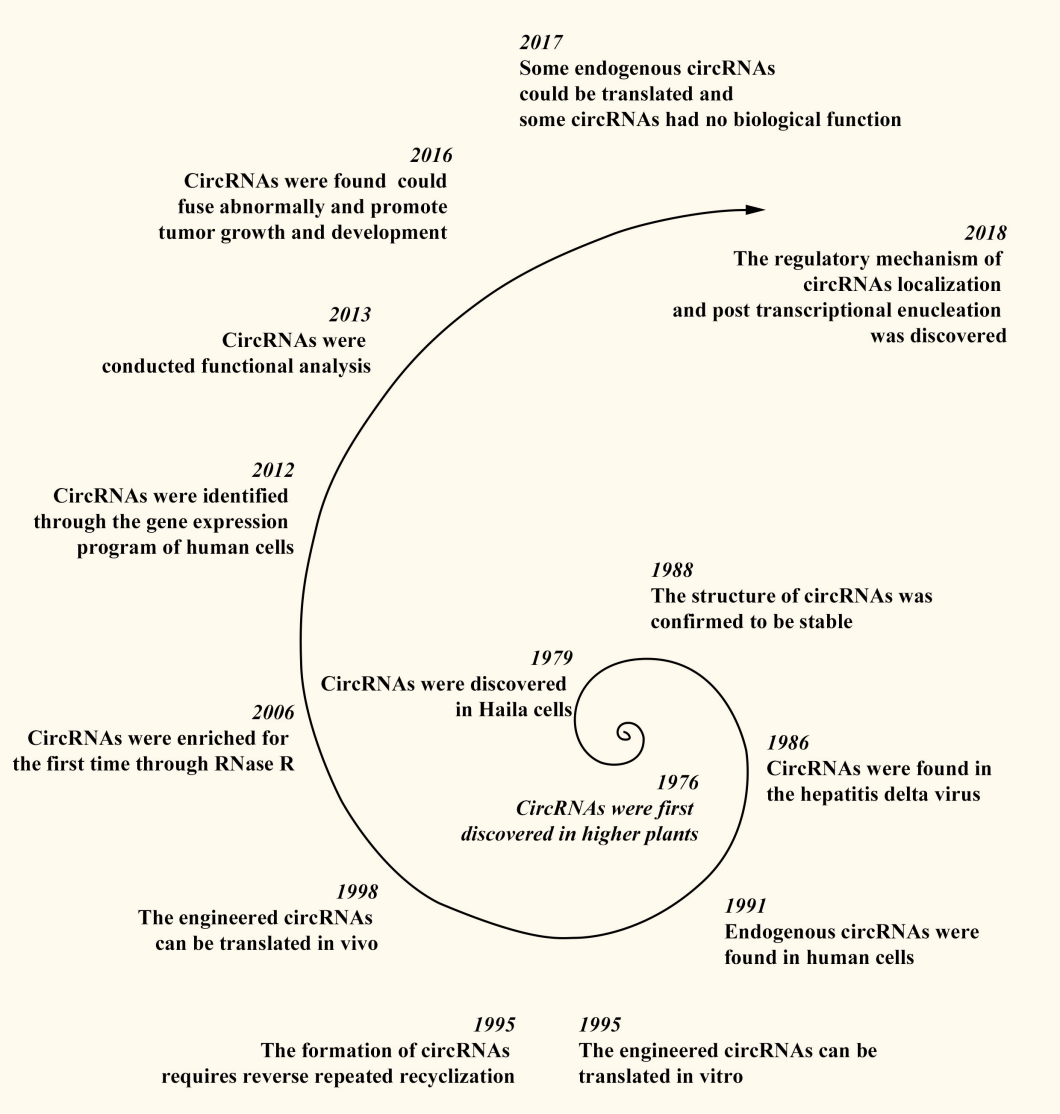
Discovery process of circRNAs.

CircRNAs were first discovered in 1976 in RNA viruses that cause diseases in higher plants (20). Subsequent research revealed additional circRNAs, with Hsu et al. identifying circRNA structures in the cytoplasm of HeLa cells in 1979 (21) and their presence in the hepatitis delta virus in 1986 (22). The stable structure of circRNAs was confirmed in 1988 (23), and endogenous circRNAs were identified in human cells in 1991 (24). Later, in 1995, Chen et al. demonstrated that engineered circRNAs can be translated *in vitro*, with the formation of circRNAs requiring reverse repeated recyclization (25, 26). In 1998, Perriman et al. confirmed that engineered circRNAs can be translated *in vivo* (27). In 2006, Suzuki et al. first enriched circRNAs using RNase R (28). Salzman et al. identified circRNAs using the gene expression program of human cells in 2012 (29). In 2013, Memczak et al. conducted functional analyses on circRNAs, providing insights into their potential roles in gene regulation (30). In 2016, researchers discovered that abnormally fused circRNAs can promote tumor growth and development (31), and in 2017, it was reported that some endogenous circRNAs can be translated, whereas others have no biological function *in vivo* (32, 33). In 2018, Huang et al. identified the regulatory mechanism of circRNA localization and posttranscriptional enucleation (34). Owing to the development of bioinformatics, over 183,000 circRNAs have been discovered in the human body (35) (**Figure 1**).

### 3.3 Characteristics of CircRNAs

#### 3.3.1 Abundance

CircRNAs are widely distributed throughout organisms and are specifically expressed and regulated in various tissues and cells (36). Furthermore, they are detected in bodily fluids such as saliva (37), plasma (38), and exocrine bodies (39).

#### 3.3.2 Specificity

The specificity of circRNAs is prominently reflected in their dynamic and context-dependent expression patterns. CircRNAs exhibit strong tissue and cell type specificity, with distinct expression profiles across various biological contexts. Additionally, their spatiotemporal regulation allows them to play roles in specific developmental stages or physiological states, while aberrant expression is often linked to disease progression, including cancer, cardiovascular disorders, and neurodegenerative diseases (40, 41). These features make circRNAs highly specific biomarkers for diagnosis and potential therapeutic targets. A study in rats revealed that, during different lactation stages, many circRNAs are specifically expressed (42).

#### 3.3.3 Stability

CircRNAs possess highly stable, closed-loop structures with connected 3’ and 5’ ends, rendering them resistant to degradation by nucleases, and their lack of 5’ cap and 3’ tail structures prevents degradation reactions such as decapping and deadenylation (43).

#### 3.3.4 Conservativeness

In terms of conservativeness, circRNAs are more conserved than their linear counterparts (44). Studies have shown that 4,522 of 15,849 circRNAs in mice have homologous sequences in humans (45), highlighting the potential translational value of circRNAs in future research.

### 3.4 Formation of CircRNAs

**Figure 2:**
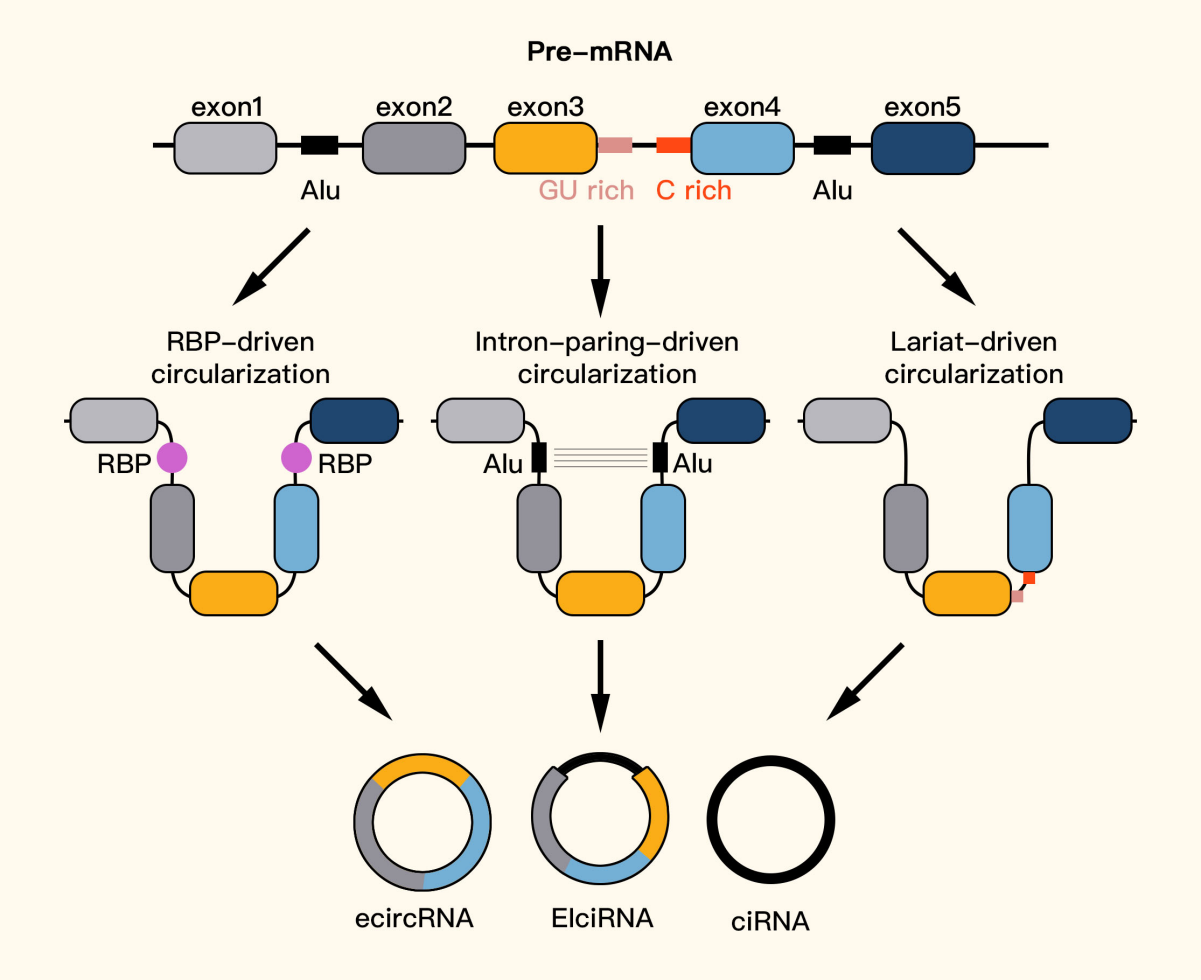
Formation of circRNAs.

CircRNAs are formed through one of three main models. The first model of circRNA formation is lasso-driven cyclization, in which partially folded pre-mRNAs cause exon hopping, connecting the downstream exon’s 3-SD (splicing donor) with the upstream exon’s 5’SA (splicing acceptor) to form an RNA lasso with both exons and introns. The introns are removed, resulting in ecircRNAs. The second model is intron-pairing-driven cyclization, which involves pairing and complementary binding between introns on the side of pre-mRNAs, forming a lasso that can either remove introns to create ecircRNAs or retain introns to form EIciRNAs. Finally, the third model involves RNA-binding protein-dependent cyclization, in which RNA-binding proteins (RBPs) bind to introns during transcription, leading to the formation of circRNAs (11, 12, 46–49). EcircRNAs are the most common type of circRNA (50) (**Figure 2**).

### 3.5 Biological functions of CircRNAs

#### 3.5.1 CircRNAs as MicroRNA sponges

MicroRNAs (miRNAs) are small ncRNA molecules, approximately 19-24 nucleotides in length (51). By binding to genes through complementary base pairing, miRNAs can inhibit mRNA translation into proteins or cause mRNA degradation (52). CircRNAs possess various miRNA response elements (MREs) that complementarily bind to miRNAs, enabling interactions such as the sponge effect, where circRNAs regulate miRNA activity (53). Hansen et al. discovered the first sponge structure of a miRNA reaction element containing 74 miR-7 (54). The most well-known circRNA that acts as a miRNA sponge is cirRS-7 (55). By inhibiting miR-7, ciRS-7 can upregulate the expression of related genes (56). CcircRNA-Sry has been found to regulate tumor progression by sponging miR-138 (13). Studies have shown that circRNAs, as miRNA sponges, play crucial roles in regulating tumor progression (57, 58).

During the progression of breast cancer, various circRNAs are activated, including upregulated circRNAs such as hsa_circ_100876, which promote metastasis by inhibiting miR-361-3p (59), and circCER, which promotes metastasis by inhibiting miR-136 (60). Additionally, the downregulation of circRNAs such as hsa_circ_000911 via the inhibition of miR-449a negatively regulates the invasion of BC (61). Moreover, circGFRA1 promotes fat phagocytosis in TNBC through miR-34a (62).

#### 3.5.2 CircRNAs regulate host gene expression

CircRNAs have the ability to directly or indirectly regulate host gene expression. For example, circRNAs containing a starting codon, such as circ_CNOT2, can regulate linear genes by forcing them to use another starting codon for translation (63). Moreover, circRNAs can promote mRNA transcription (64) and are indirectly regulated by increasing the activity of RNA polymerase II (65). Li et al. reported that EIcRNAs can interact with nuclear ribonucleoprotein (U1snRNP) to promote host gene transcription (66). Additionally, SOX8 is involved in the maintenance of stem-like capacities in TNBC cells (67).

#### 3.5.3 Interactions between CircRNAs and proteins

RBPs are crucial for posttranscriptional regulation, including mRNA splicing, stabilization, localization, modification, and translation (68). CircRNAs can interact with RBPs, altering the function of related proteins (69). For example, circCCNB1 can interact with cyclin-dependent kinase 1 and cyclin B1, affecting p53 gene mutation in breast cancer (70). Circ-PABPN1 can bind to the RBP HuR, hindering PABPN1 translation (71). Moreover, some circRNAs can act as scaffold molecules for protein−protein interactions. For example, circAmotl1 can bind to protein kinase B (AKT1) and inositol 3-phosphate-dependent protein kinase 1 (PDK1) (72).

#### 3.5.4 CircRNAs participate in the immune response

CircRNAs reshape the tumor microenvironment (TME) by regulating epithelial–stromal transition, tumor angiogenesis, immune cell function, and the inflammatory response. Immune cells are the most abundant TME components and play a critical role in breast cancer cell progression (73). CircRNAs have been shown to promote cellular responses to external stimuli by binding to specific proteins. This is particularly evident in the rapid generation of immune responses following viral infections (74). Furthermore, some exogenous circRNAs can recognize the receptor RIG-1, consequently stimulating immune signal transduction in mammalian cells (75). CircRNAs in the TME can increase the expression of immune checkpoint molecules, including PD-L1, PD-1 and CD73, on the surface of tumor cells through miRNA sponge action and help tumor cells escape the toxic killing of immune cells (76).

#### 3.5.5 CircRNAs participate in protein translation

Although circRNAs lack 3’ and 5’ end caps, experiments have revealed that artificial circRNAs containing open reading frames for green fluorescent protein can be translated into these proteins in E. coli (77). Some circRNAs containing open reading frames can participate in protein translation (11). CircRNAs are primarily involved in protein translation via the N6-methyladenosine-dependent pathway and the IRES-dependent pathway (78). They drive initial transcription and protein translation through base-modified N6 methyl adenosine (m6A) sites (79). CircRNAs with IRES sites are instrumental in protein translation after binding to ribosomes (80). Proteins translated by circRNAs potentially possess antitumor properties by blocking tumor metastasis or metabolism (81).

## 4 Progress of CircRNAs in breast cancer

### 4.1 Expression of CircRNAs in breast cancer

**Figure 3:**
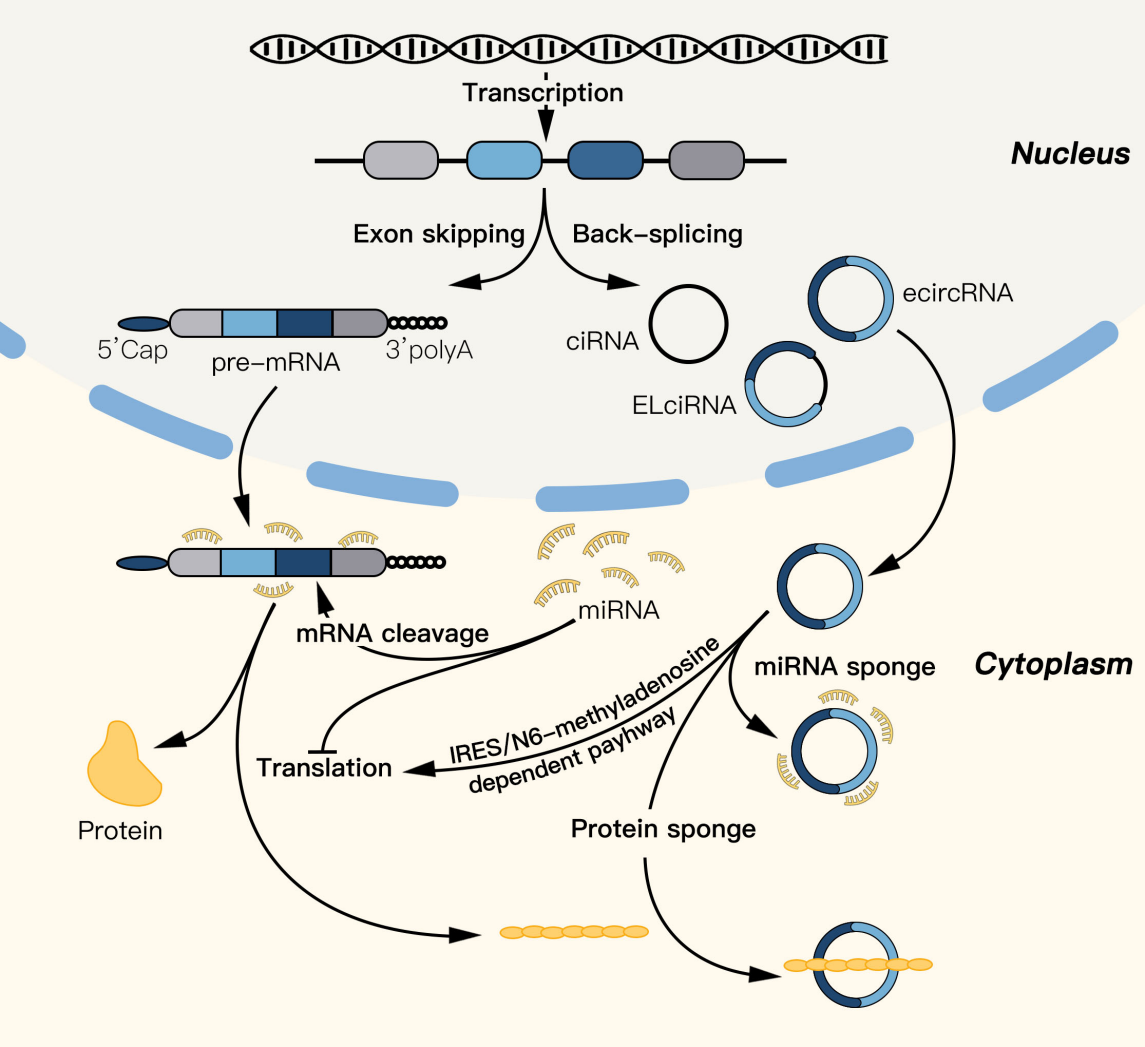
The main biological mechanisms of circRNAs.

CircRNAs significantly impact the progression of BC by influencing various cellular processes, such as proliferation, invasion, apoptosis, and drug resistance (82). Wang et al. demonstrated that estrogen-induced circPGR plays a crucial role in ER-positive BC through circRNA sequencing (83). High-throughput sequencing has revealed a vast number of differentially expressed circRNAs in BC tissue compared with healthy tissue (84). Through gene chip analysis, 19 upregulated and 22 downregulated circRNAs were identified in breast cancer tissues compared with normal tissues (85). Specific circRNAs, such as circKIF4A, which is correlated with the survival rate of patients with TNBC (86), are upregulated, and circMYO9B is correlated with prognosis (87). Conversely, some circRNAs, such as circTADA2As, are downregulated, which is associated with the survival rate of patients with TNBC (88), and circ-NOL10, which is downregulated in breast cancer tissue (89) (**Figure 3**).

### 4.2 Mechanisms of CircRNAs in breast cancer

#### 4.2.1 CircRNAs regulate the regeneration and proliferation of breast cancer stem cells

Breast cancer stem cells have been identified as major factors in the onset of breast cancer (90). These cells are also responsible for the frequent metastasis and recurrence of the disease because of their ability to promote tumor growth and their resistance to conventional treatments (91). Accordingly, research has identified breast cancer stem cells as potential targets for BC treatment (92, 93). Yan et al. hypothesized that circRNAs could act as miRNA sponges, influencing the regulation of breast cancer stem cell proliferation and renewal (94). Lin et al. discovered that TV-CircRGPD6 targets circRGPD6, which results in increased expression of p-H2AX, a DNA damage marker, and reduced expression of CD44, a stem cell marker (95). Additionally, circ_002178 overexpression promotes increased ALDH1 activity and increases the expression of stem cell markers in SUM149PT cells (96).

#### 4.2.2 CircRNAs regulate the cell cycle and proliferation in breast cancer

One of the circRNAs shown to play a role in BC is circRNF10-DHX15. This circRNA inhibits the proliferation of cancer cells by sponging the DHX15-NF-κB p65 positive feedback loop (97). Another circRNA, circZFAND6, which is a sponge of miR-647, promotes cell migration and proliferation in breast cancer (98). CircRNAs can also regulate the cell cycle and proliferation of BC through cancer-related signaling pathways. For example, circRNA-069718 promotes cell proliferation and invasion in TNBC via the Wnt activation/β-catenin pathway (99), whereas circ-ITCH inactivates the Wnt/β-catenin signal, inhibiting cell proliferation in TNBC (100).

#### 4.2.3 CircRNAs regulate the epithelial mesenchymal transformation, invasion and metastasis of breast cancer

EMT is a process in which epithelial cells transform into a mesenchymal phenotype through a specific program. As a result, a reduction in adhesion between tumor cells promotes tumor invasion and metastasis (101). EMT is known to cause tumorigenic changes in the tumor microenvironment (102). Several circRNAs have been shown to regulate EMT in BC. For example, circROBO1 promotes breast cancer carcinogenesis and liver metastasis via the circROFO1/KLF5/FUS feedback loop. Furthermore, circROBO1 inhibits the transcription of BECN1 and prevents the selective autophagy of afadin (103). Leng et al. reported that CIRC_0000043 regulates the miR-136/Smad3 axis to promote the EMT, invasion and metastasis of breast cancer (104). Several studies have examined the role of specific circRNAs in breast cancer metastasis. Song et al. reported that circHMCU targets EMT and G phase cell cycle checkpoints, thereby promoting the progression of BC (105). Additionally, the upregulation of circ_IRAK3 (hsa_circular RNA_0005505) in metastatic breast cancer is related to the recurrence of distant organs (106). Furthermore, Mao et al. reported that circRNA_000554 binds to miR-182 and suppresses epithelial stromal transformation in breast cancer (107). Conversely, Pan et al. demonstrated that the CIRC-TFF1 gene regulates the miR-326/TFF1 axis, thereby inhibiting the progression of BC (108).

#### 4.2.4 CircRNAs are related to the tumor microenvironment and the immune escape of breast cancer

Breast cancer cells thrive in a hypoxic tumor microenvironment, and one circRNA, HIF1α-associated circDENND4C, is known to promote their proliferation by combining with miR-200b and miR-200c, promoting glycolysis, invasion, and metastasis (109, 110). Li et al. reported that hsa_circ_0067842 is expressed in breast cancer, which promotes immune escape by stabilizing CMTM6 via HuR, subsequently inhibiting PD-L1 ubiquitination and degradation ultimately leading to increased PD-L1 expression and enhanced tumor immune evasion (111).

#### 4.2.5 CircRNAs regulate apoptosis in breast cancer

The most upregulated circRNA among the candidate genes in TNBC cells, circ-EPSTI1, can inhibit apoptosis, whereas knockout of circ-EPSTI1 can induce apoptosis (112). In addition, circ-FOXO3 is downregulated in BC cells but upregulated during apoptosis (113).

#### 4.2.6 CircRNAs regulate the breast tumor immune microenvironment

Many circRNAs have been shown to regulate the tumor immune microenvironment and cause immune escape of tumor cells (114). Several circRNAs have been proven to be related to the tumor immune microenvironment in breast cancer. Circ_0001142 is highly expressed in breast cancer cells, and the circ_0001142/miR-361-3p/PIK3CB pathway is involved in the polarization and autophagy of macrophages (115). In addition, circRNAs can regulate T-cell activity and immune checkpoint molecules. The circular RNA circWWC3 can increase IL-4 expression and secretion in breast cancer cells, which further enhances the expression of PD-L1 and facilitates breast cancer immune evasion (116). Ectopic circ_002172 expression could inhibit cytotoxic T lymphocyte infiltration in breast cancer and promote immune escape (117). CircRNAs have also been found to be associated with macrophages in breast cancer. Circ-Ccnb1 interacts with wild-type p53 and allows Bclaf1 to bind Bcl2, resulting in the induction of cell death (70). The circular RNA CDR1as/ciRS-7 plays an essential role in the immune microenvironment of breast cancer, including M2 macrophages. CDR1as regulates the TGF-β signaling pathway and ECM-receptor interaction to contribute to the immune microenvironment of breast cancer (118). These findings identify several circRNAs as diagnostic biomarkers and potential targets for breast cancer therapy.

### 4.3 Clinical correlation between CircRNAs and breast cancer

#### 4.3.1 CircRNAs can be used as potential biomarkers for the diagnosis, staging, prognosis of breast cancer and therapeutic target

CircRNAs are promising biomarkers for liquid biopsy because of their wide distribution in the body, stable structure, high sensitivity, and strong specificity (119, 120). There is a link between circUSP42 downregulation and the late clinical stage and lymph node metastasis of TNBC (121). The plasma concentration of hsa_circ_0001785 decreases significantly after surgical removal of breast cancer masses, suggesting its potential as a biomarker for evaluating prognosis (85). This may be attributed to the decrease in the amount of tumor-derived nucleic acid released after surgery (122). The overexpression of circWWC3 activates the RAS signaling pathway, which is associated with poor prognosis in BC patients (123).

Owing to their demonstrated association with cancer progression, multiple circRNAs have been identified as potential therapeutic targets for BC (124). CircRAD18 promotes the progression of TNBC by functioning as a miR-208a/3164 sponge to regulate IGF1 and FGF2 expression. Therefore, circRAD18 is a therapeutic target for TNBC (125). Through its involvement in the miR-675/NEDD4L axis, circKDM4B impedes breast cancer progression, particularly in terms of angiogenesis and tumor metastasis. Given these findings, circKDM4B has significant potential as a therapeutic target (126).

#### 4.3.2 CircRNAs affect chemoresistance of breast cancer

Chemotherapy reduces cancer metastasis and recurrence and ensures successful surgery. However, chemotherapy resistance arising from long-term use is the primary reason for breast cancer treatment failure (127). Common chemotherapy medications include adriamycin, cyclophosphamide, doxorubicin, paclitaxel, and 5-fluorourazalidine (128). Through the miR-361-5p/TLR4 pathway, circGFRA1 reduces TNBC cell sensitivity to paclitaxel (PTX) (129), whereas circ_0006528 promotes the progression of paclitaxel-resistant breast cancer cells. Nevertheless, it is possible to inhibit the growth of drug-resistant cells by silencing circ_0006528 (130). CircKDM4C binds to miR-548p and reduces breast cancer progression, thus slowing drug resistance to doxorubicin (131). Furthermore, circUBE2D2 disruption inhibits the invasion of TNBC and decreases doxorubicin resistance (132). Finally, studies have suggested that the downstream molecule Rafl of the hsa_circ_0006528/miRNA-7-5p axis influences BC cell resistance to doxorubicin (133).

### 4.4 The latest research on CircRNAs in TNBC

#### 4.4.1 Mechanism of CircRNAs in TNBC

CircRNAs affect the progression of TNBC, primarily as miRNA sponges. For example, Chen et al. discovered that estrogen receptor β2 (ERβ2) is responsible for mediating hsa_cir_0000732, acting as a sponge of miR-1184 and promoting the migration, growth and invasion of TNBC (134). Gong et al. reported that circUBR5 maintains the malignant growth of TNBC through miR-1179 absorption and UBR5 upregulation both *in vitro* and *in vivo* (135). According to Shao et al., circ_0004676 regulates miR-377-3p/E2F6/PNO1, playing a carcinogenic role in TNBC (136). In addition, hsa_cir_102229 regulates the expression of PFTK1 through combining with miR-152-3p, hence impacting TNBC cell function (137). In a study by Li et al., circ_0041732 promoted TNBC invasion and metastasis and regulated tumor characteristics via miR-149-5p/FGF5 (138). Finally, circFAM64A targets the 3’UTR of Cdc10-dependent transcript 1 (CDT1), and elevated CDT1 expression often correlates with a dismal prognosis (139). The circular RNA CDR1as/ciRS-7 has a comprehensive role in interacting with various immune cells, including T cells, NK cells and macrophages, and leads to TME reshaping and breast cancer progression (118).

#### 4.4.2 Biological function of CircRNAs in TNBC

Differential expression of circRNAs in TNBC and adjacent tissues has been previously observed. Magalhães et al. reported that 16 kinds of circRNAs were differentially expressed between TNBC patients and control individuals. Some circRNAs interact with RBPs involved in cancer and gene regulation pathways, including PTBP1, ELAVL1, EIF4A3, and AGO1/2 (140).

CircRNAs also impact cell cycle and proliferation of TNBC cells. For example, IGF2BP2 and HuR use circEIF3H as a scaffold to promote TNBC proliferation and metastasis, as reported by Song et al. (141). Additionally, Barznegar et al. reported that the upregulation of circ-ELP3 promotes TNBC development (142). The authors showed that circ_0076611 interacts with proliferation-related transcripts, thereby amplifying the progression of TNBC (143). In addition, Ruan et al. reported that circMETTL3 acts as a miR-34c-3p sponge to inhibit TNBC invasion and metastasis (144).

In TNBC, CircRNAs play a significant role in epithelial mesenchymal transformation, invasion, and metastasis. For example, overexpression of circBACH2 induces TNBC cell proliferation and epithelial mesenchymal transformation (145). CircCD44 promotes TNBC proliferation and invasion via the miR-502–5p/KRAS and IGF2BP2/Myc pathways (146). Zan et al. demonstrated that by targeting the miR-28-5p/LDHA pathway, circ-CSNK1G1 promotes TNBC cell invasion, metastasis, and glycolysis (147). CircPRKCI, as a miR-545-3p sponge, regulates the phosphorylation of WBP2 and AKT to increase TNBC migration (148). Via the miR-136–5p/PDK4 pathway, circERBB2 facilitates the Warburg effect and increases the growth of TNBC (149). Furthermore, the downregulation of circPTK2 promotes TNBC proliferation and invasion (150). Additionally, the knockdown of some circRNAs, such as circ_0062558, affects the miR-876-3p/SLC1A5 axis (151), circ_000520 affects the miR-1296/ZFX axis (152), and circDHDDS affects the miR-362-3p/DDX5 axis (153), induces the apoptosis of TNBC cells.

#### 4.4.3 Clinical correlation between CircRNAs and TNBC

As a potential prognostic biomarker for TNBC, circNR3C2, combined with miR-513a-3p, inhibits HRD1-mediated tumor growth (154). Xing et al. reported that circ-PDCD11 overexpression is an independent risk factor associated with poor prognosis in TNBC patients (155). Similarly, circ-TRIO regulates miR-432-5p/CCD58 and can be a new prognostic marker for TNBC, as reported by Wang et al. (156). Moreover, Li et al. demonstrated that both the Wnt/beta-catenin pathway and MYH9 stabilization promote the progression of TNBC and that circ-EIF6-encoded EIF6-224a is involved in this process (157). These findings suggest that targeting circ-EIF6/EIF6-224 aa could be an approach for both prognostic and therapeutic purposes in TNBC treatment. CircPSMA1 can activate the miR-637/Akt1/β-catenin (cyclin D1) axis, promoting TNBC tumorigenesis and metastasis. These findings highlight the potential of circPSMA1 as a biomarker and target for TNBC immunotherapy (158). Chen et al. reported that circHIF1A regulates NFIB expression and translocation to promote TNBC progression. Additionally, circHIF1A is upregulated in the plasma, making it a potential target for TNBC diagnosis and treatment (159). The circRNA hsa_cir_0006220 has been found to inhibit tumor growth in TNBC by regulating miR-197-5p/CDH19, making it another promising therapeutic target (160). Furthermore, CircRAD54L2 modulates the miR-888 family/PDK1 axis to promote TNBC proliferation, invasion, and metastasis, making it another potential therapeutic target in TNBC (161).

CircRNAs can also affect the chemoresistance of TNBC. For example, cirCWAC induces chemoresistance by targeting miR-142, activating the PI3K/AKT pathway, and upregulating WWP1 (162). The circRNA CREIT reduces the stability of PKR and thus improves the drug resistance of TNBC to doxorubicin (163); moreover, CircINTS4 promotes the chemoresistance of TNBC through competitive binding with the miR-129-5p/POM121 axis (164). CircUBAP2, as a sponge of miR-300, upregulates ASF1B and triggers PI3K/AKT/mTOR (PAM) signaling to increase TNBC resistance to cisplatin (165) (**Table 3**).

**Table 3:** Conclusion on the functions of circRNAs in breast cancer.

circRNA	Up/Down	Target	Functions	Reference
hsa_circ_100876	Up	microRNA-361-3p	Oncogene	(59)
circRNA-CER	Up	miR-136	Oncogene	(60)
hsa_circ_000911	Down	miR-449a	Tumor Suppressor	(61)
circGFRA1	Up	miR-34a	Oncogene	(62)
circKIF4A	Up	miR-375	Oncogene	(86)
circMYO9B	Up	miR-4316	Oncogene	(87)
circTADA2As	Down	miR-203a-3p	Tumor Suppressor	(88)
circ-NOL10	Down	miR-149-5p	Tumor Suppressor	(89)
circ-NOL10	Down	miR-330-3p	Tumor Suppressor	(89)
circ-NOL10	Down	miR-452-5p	Tumor Suppressor	(89)
circRGPD6	Down	miR-26b	Tumor Suppressor	(95)
circ_002178	Up	miR-1258	Oncogene	(96)
circRNF10	Down	DHX15	Tumor Suppressor	(97)
circZFAND6	Up	miR-647	Oncogene	(98)
circRNA-069718	Up	Wnt/β-catenin pathway	Oncogene	(99)
circ-ITCH	Down	miR-214 and miR-17	Tumor Suppressor	(100)
circROBO1	Up	miR-217-5p	Oncogene	(103)
circ_0000043	Up	miR-136	Oncogene	(104)
circHMCU	Up	let-7 Family	Oncogene	(105)
circIRAK3	Up	miR-3607	Oncogene	(106)
circRNA_000554	Down	miR-182	Tumor Suppressor	(107)
circ_0061825	Down	miR-326	Tumor Suppressor	(108)
circDENND4C	Up	miR-200b/c	Oncogene	(110)
hsa_circ_0067842	Up	HuR	Oncogene	(111)
circEPSTI1	Up	miR-4753 and miR-6809	Oncogene	(112)
circ-FOXO3	Down	p53 and MDM2	Tumor Suppressor	(113)
circ_0001142	Up	miR-361-3p	Oncogene	(115)
circWWC3	Up	miR-26b-3p and miR-660-3p	Oncogene	(116)
circUSP42	Down	miR182	Tumor Suppressor	(121)
circRAD18	Up	miR-208a/3164	Oncogene	(125)
circKDM4B	Up	miR-675	Oncogene	(126)
circGFRA1	Up	miR-361-5p	Oncogene	(129)
circ_0006528	Up	miR-1299	Oncogene	(130)
circKDM4C	Down	miR-548p	Tumor Suppressor	(131)
circUBE2D2	Up	miR-512-3p	Oncogene	(132)
hsa_circ_0006528	Up	miRNA-7-5p	Oncogene	(133)
hsa_cir_0000732	Up	miR-1184	Oncogene	(134)
circUBR5	Up	miR-1179	Oncogene	(135)
circ_0004676	Up	miR-377-3p	Oncogene	(136)
hsa_cirRNA_102229	Up	miR-152-3p	Oncogene	(137)
circ_0041732	Up	miR-149-5p	Oncogene	(138)
circFAM64A	Up	miR-149-5p	Oncogene	(139)
circEIF3H	Up	IGF2BP2/HuR	Oncogene	(141)
circ-ELP3	Up	ELP3 mRNA	Oncogene	(142)
circ_0076611	Up	VEGFA	Oncogene	(143)
circMETTL3	Down	miR-34c-3p	Tumor Suppressor	(144)
circBACH2	Up	miR-186-5p/miR-548c-3p	Oncogene	(145)
circCD44	Up	miR-502-5p and IGF2BP2	Oncogene	(146)
circ-CSNK1G1	Up	miR-28-5p	Oncogene	(147)
circPRKCI	Up	miR-545-3p	Oncogene	(148)
circ-ERBB2	Up	miR-136-5p	Oncogene	(149)
circPTK2	Down	miR-136	Oncogene	(150)
circ_0062558	Up	miR-876-3p	Oncogene	(151)
circ_000520	Up	miR-1296	Oncogene	(152)
circDHDDS	Up	miR-362-3p	Oncogene	(153)
circNR3C2	Down	miR-513a-3p	Tumor Suppressor	(154)
circ-PDCD11	Up	miR-432-5p	Oncogene	(155)
circ-TRIO	Up	miR-432-5p	Oncogene	(156)
circ-EIF6	Up	Wnt/beta-catenin pathway	Oncogene	(157)
circPSMA1	Up	miR-637	Oncogene	(158)
circHIF1A	Up	NFIB	Oncogene	(159)
hsa_cir_0006220	Down	miR-197-5p	Tumor Suppressor	(160)
CircRAD54L2	Up	miR-888	Oncogene	(161)
cirCWAC	Up	miR-142	Oncogene	(162)
circRNA-CREIT	Up	PKR	Oncogene	(163)
circINTS4	Up	miR-129-5p	Oncogene	(164)
circUBAP2	Up	miR-300	Oncogene	(165)

## 5 Discussion

### 5.1 Challenges of CircRNAs in Breast Cancer Research

1. Current studies have focused primarily on the role of circRNAs as miRNA molecular sponges in breast cancer, with less attention given to their interactions with other potential molecules, such as proteins or other RNA types. The biological functions of circRNAs and their role in regulating the breast tumor microenvironment remain incompletely understood. Furthermore, the clinical relevance of circRNAs in breast cancer, particularly with respect to chemotherapy resistance, has yet to be fully elucidated.

2. Some circRNAs undergo nuclear-to-cytoplasmic localization conversion, but the mechanisms underlying this process and their nuclear export remain poorly understood. Additionally, while circRNAs are characterized by stable closed-ring structures, their biogenesis and degradation mechanisms require further investigation.

3. Owing to their stability, abundance, and tissue specificity, circRNAs hold great potential in cancer diagnosis, prognosis, and treatment. They can serve as noninvasive biomarkers and therapeutic targets by regulating key pathways, such as miRNA sponging and drug resistance. Synthetic circRNAs or delivery systems could restore tumor-suppressing functions or counteract oncogenic signals. Additionally, their role in immune modulation offers prospects for enhancing immunotherapies. With further clinical validation, circRNAs could revolutionize precision medicine and address challenges such as drug resistance in cancer treatment. However, applying these findings to clinical practice presents a significant challenge.

## 6 Conclusion

Currently, research on circRNAs in breast cancer is an emerging field, and future directions can focus on the following areas:

1. Clarify circRNA biogenesis, cellular localization, and degradation mechanisms.
2. Investigate additional “sponge” effects of circRNAs on miRNAs and elucidate the mechanisms behind other biological functions of circRNAs.
3. Identify the mechanisms by which circRNAs interact with multiple immune cell types, reshape the breast TME, regulate breast cancer cells, and provide potential therapeutic targets.
4. Examine and identify the functions of circRNAs in BC, particularly their role in contributing to drug resistance in breast cancer treatments beyond chemotherapy (e.g., radiotherapy and immunotherapy).
5. Further screen and verify circRNA biomarker candidates and therapeutic targets are needed, with an emphasis on early clinical application.
6. Develop a standardized classification method for circRNA sources, distribution, functions, and naming conventions.
